# TRAJECTORIES OF EPISODIC MEMORY IN MIDLIFE: HISTORICAL CHANGE FROM A CROSS-COUNTRY PERSPECTIVE

**DOI:** 10.1093/geroni/igae098.2198

**Published:** 2024-12-31

**Authors:** Markus Wettstein, Frank Infurna, Nutifafa Dey, Yesenia Cruz-Carrillo, Kevin Grimm, Margie Lachman, Denis Gerstorf

**Affiliations:** Humboldt University Berlin, Berlin, Berlin, Germany; Arizona State University, Tempe, Arizona, United States; Arizona State University, Tempe, Arizona, United States; Arizona State University, Tempe, Arizona, United States; Arizona State University, Tempe, Arizona, United States; Brandeis University, Waltham, Massachusetts, United States; Humboldt University Berlin, Berlin, Berlin, Germany

## Abstract

According to the Flynn effect, cognitive abilities have improved across the past decades. However, we know little about whether such historical improvements generalize to middle-aged adults and differ across nations. In this study, we used harmonized data on episodic memory from nationally representative longitudinal panel surveys including the U.S., Europe, Mexico, and China to compare historical change in age-related trajectories of episodic memory among middle-aged adults. Our sample included 89,775 participants aged 45 to 65 years who provided 272,876 observations over up to 20 years. Longitudinal multilevel regression models revealed that today’s middle-aged adults in the U.S. perform worse on episodic memory tests than their age peers in the past. In contrast, today’s middle-aged adults in most other countries perform better than did their peers in the past. At the same time, later-born cohorts of U.S. middle-aged adults exhibited a more favorable episodic memory trajectory than earlier-born cohorts, whereas this trend was not observed in most other countries. Cohort trends remained significant when controlling for socio-demographic indicators (gender, education) and health measures (grip strength, number of chronic diseases). Women and individuals with higher levels of education, higher grip strength and fewer chronic diseases exhibited better episodic memory performance, and the association of grip strength with episodic memory was stronger in later-born cohorts. Our findings suggest that countries differ in the extent and direction of historical change in episodic memory scores and trajectories. More research is needed to better understand why the Flynn effect in the U.S. seems to be reversed.

